# A European, multicentre, observational, post-authorisation safety study of oral sulphate solution: compliance and safety

**DOI:** 10.1055/a-1090-7289

**Published:** 2020-02-21

**Authors:** Jaroslaw Regula, Manon C.W. Spaander, Stepan Suchanek, Anne Kornowski, Valerie Perrot, Wolfgang Fischbach, the DUS investigators

**Affiliations:** 1 Medical Centre for Postgraduate Education and Maria Sklodowska-Curie Institute-Oncology Centre, Warsaw, Poland; 2 Department of Gastroenterology and Hepatology, Erasmus MC Cancer Institute, Rotterdam, The Netherlands; 3 Department of Internal Medicine, 1st Faculty of Medicine, Charles University, Military University Hospital, Prague, Czech Republic; 4 Ipsen Pharma, Boulogne-Billancourt, France; 5 Medizinische Klinik II, Klinikum Aschaffenburg-Alzenau, Aschaffenburg, Germany

## Abstract

**Background and study aims** Oral sulphate solution (OSS) is a sulphate-based, low-volume bowel cleansing preparation taken in two doses of 500 mL, each followed by 1000mL of water or clear liquid. The primary objective of this observational study was to document compliance with the recommended hydration guidelines in a representative sample of the European population.

**Patients and methods** Prospective, non-interventional, multicentre study (NCT02630680, EUPAS9361) in patients prescribed OSS for colonoscopy preparation in routine clinical practice in Europe. Patients were included according to pre-agreed consecutive enrolment rules. Patients recorded the volume of OSS and water or clear liquid intake, and occurrence of adverse events (AEs). Compliance with hydration was calculated as a ratio of actual volume of water/clear liquid taken versus prescribed 2,000 mL, and non-compliance defined as < 75 % intake. Colon cleansing level was assessed on a 4-point scale.

**Results** Between October 2015 and January 2017, 1,281 patients were recruited in 16 centres in four European countries (safety population n = 1,206; registry population n = 1,177). Of patients, 94.5 % were ≥ 75 % and 86.8 % 100 % compliant with hydration guidelines. Patients took an average of 96.8 % of the recommended OSS volume; 46 patients (3.9 %) were non-compliant. Colon cleansing levels were good-to-excellent in 87.6 % of patients. Three hundred and twenty-nine patients (27.3 %) experienced 758 treatment-related AEs, mostly gastrointestinal (82.9 %), all were mild-to-moderate. Non-compliant patients had no AEs suggestive of dehydration.

**Conclusion** In this non-interventional study in a real-life setting, treatment compliance with hydration guidelines was good-to-excellent in 94.5 % of patients receiving OSS. The safety profile of OSS was similar to the prescribing information.

TRIAL REGISTRATION: Observational study EUPAS9361 at www.encepp.eu

## Introduction

Colonoscopy plays an important role in diagnosis and management of colorectal diseases, and it is the current standard method for early detection and treatment of colorectal precancerous lesions and colorectal cancer (CRC) 1 . Effective bowel cleansing is mandatory to ensure good visualisation of the entire colonic mucosa during colonoscopy and for detection and removal of all suspicious precancerous lesions in asymptomatic individuals 2 . The quality of colonic cleansing is therefore a key factor influencing the diagnostic accuracy of colonoscopies 3 4 . Suboptimal bowel preparation not only leads to missed colonic lesions (especially smaller lesions), but it is also associated with increased costs due to rescheduling and wasted resources as a follow-up examination is required in such cases 5 6 . Inadequate colon cleanliness at colonoscopy has been reported in up to 30 % of patients 1 .

While patient-specific factors, comorbidities and co-medications may influence the quality of the bowel preparation for colonoscopy 7 8 9 , compliance with bowel cleansing preparation is a key factor for optimal bowel cleansing 2 , and patient willingness to complete the full preparation is an important determinant of the quality of bowel cleansing. A low volume of solution, with an acceptable taste 10 , given in a split dose regimen (i. e., night before and morning of procedure) 11 12 appears to be the most acceptable and effective of all preparations 13 .

Oral Sulphate Solution (OSS; Eziclen/Izinova; Ipsen Pharma, France) is a low-volume bowel cleansing preparation administered as two doses of 500 mL, each followed by 1000 mL of water or clear liquid for hydration. OSS was approved in Europe in January 2013, either as a split-dose or day-before dosing regimen, and is indicated in adults for bowel cleansing before any procedure requiring a clean bowel (e. g., bowel visualisation prior to endoscopy/radiology or a surgical procedure). The post-approval commitments for OSS in Europe included the request that Ipsen Pharma conduct a study to assess drug utilisation in the real-life setting in a representative sample of the European target population. Studies in a real-life setting can be beneficial to detect rare adverse events (AEs), differences in effects in sub-populations, and to observe compliance in routine practice. This study was conducted to document non-compliance with the prescription hydration guidelines and to describe the safety profile of OSS (with a specific analysis of the safety profile in non-compliant patients and special populations such as the elderly and people at risk of electrolyte shifts), in the real-life setting in the post-approval period in four European countries where the product was marketed at the time of study initiation. The setting of bowel cleansing before colonoscopy was selected as it is the most frequent indication for bowel cleansing.

## Patients and methods

This was a prospective, non-interventional, multicentre study in patients receiving OSS before colonoscopy for different indications in routine clinical practice (ClinicalTrials.gov NCT02630680, EU PAS registry number EUPAS9361), requested by the European Medical Agency as a mandatory post-authorisation safety study (PASS). The study was approved by the Pharmacovigilance Risk Assessment Committee (PRAC), and the protocol and redacted abstract are available on the European Network of Centres for Pharmacoepidemiology and Pharmacovigilance ( www.ENCePP.com , ID 30186).

Inclusion began on October 12, 2015 and ended on January 20, 2017. The study was conducted at 16 centres in the Czech Republic, Germany, the Netherlands and Poland and was declared to the relevant Independent Ethics Committee (IEC)/Institutional Review Board (IRB). The study was conducted in compliance with the IEC/IRB, informed consent regulations, the declaration of Helsinki and the GEP/GPP guidelines 14 . Written informed consent for use of a patient’s sensitive data was obtained from the patient, or their legally acceptable representative, before entry into the study.

### Patients

Patients who were eligible for OSS prescription before colonoscopy (for any indication) according to the Eziclen/Izinova approved marketing authorisation (in accordance with the Summary of Product Characteristics) 15 , and who signed the informed consent form, were included at each participating centre. Exclusion criteria were non-eligibility or contraindications for Eziclen/Izinova according to the Summary of Product Characteristics (including congestive heart failure, severe renal insufficiency, inflammatory bowel disease), refusal to provide informed consent to study participation, or the prescription of bowel cleansing formulations other than Eziclen/Izinova by the physician. To minimize bias, the protocol required the inclusion of consecutive patients, but to minimise disruption to daily activities at each participating centre, investigators could space inclusions at regular intervals according to pre-agreed consecutive enrolment rules (e. g., 15 patients maximum per week, or only on Mondays and Fridays). To facilitate inclusion of patients at risk of electrolyte shifts, hospital centres as well as endoscopy clinics were included.

### Assessments and outcomes

The study took place over two visits for each patient. At the first visit when patients were included in the study and gave written informed consent, information was collected on patient characteristics (age and gender), vital signs (blood pressure, heart rate, height and weight), medical and surgical history, physical examination, the indication for bowel preparation and the OSS dosing regimen prescribed (one day or split dose regimen), and current or previous (in the last month) comedications. If laboratory test or electrocardiogram (ECG) results were available from within 7 days of this visit, these were recorded. At this visit, patients were provided with a patient diary/leaflet and were instructed to record compliance with hydration, preparation intake and dietary recommendations, and AEs.

The second visit was conducted at the time of colonoscopy and involved physical examination (including vital signs) and recording information on current or previous comedications. If a patient presented with clinical signs of dehydration, including vital sign abnormalities, these were recorded as AEs. The patient diary/leaflet ( **Appendix 2** ) was collected and AEs occurring from the first visit to discharge after colonoscopy, and patterns and conditions of OSS use, were recorded. Compliance with hydration was calculated as a ratio of actual volume taken versus the prescribed hydration volume (2000 mL), classified as excellent (100 % compliance), good (≥ 75 % and < 100 % compliance), low (≥ 50 % and < 75 % compliance), or bad (< 50 % compliance). Compliance was assessed from the patients’ estimate of the remaining volume of OSS and additional water/clear liquid. This was measured using a dosing cup that is provided with the preparation. Non-compliance was defined as having taken < 75 % of hydration. Missing volumes were imputed as not taken. The dosing schedule was calculated retrospectively from the timing of doses recorded in the patient diary. This visit also included an investigators’ assessment of colon cleansing level, which was scored on a 4-point scale (non-validated, but used in previous studies including the registration study for Eziclen/Izinova 16 17 18 19 20 ) as poor (large amounts of faecal residue, additional cleansing required), fair (enough faeces or fluid to prevent a completely reliable examination), good (small amounts of faeces or fluid not interfering with examination) or excellent (no more than small bits of adherent faeces/fluid).

The primary endpoint was the proportion of participants in the registry population that were non-compliant with the recommended hydration (i. e., took < 75 % of the 2000 mL hydration fluid). Secondary endpoints included: the proportion of participants in the registry population with excellent, good, low and bad compliance with hydration; compliance with the recommended volume of solution intake, the proportion of participants in the complete population achieving investigator-assessed cleansing level of excellent, good, fair and bad; and AEs (coded using MedDRA version 18.1; serious AEs [SAEs] were defined using the ICH 2A definition) reported in the safety population. Endpoints were also reported in predefined special populations consisting of the elderly (aged ≥ 65 years) and people at risk of electrolyte shifts but not contraindicated for OSS (patients with suspected liver disease/hepatic insufficiency, hyperuricaemia or history of gout and renal insufficiency), and patients with inflammatory bowel disease.

### Statistics

The planned sample size was 1,285 patients. The sample size was based upon the assumption that the special populations would represent 30 % of recruited patients; populations of previous clinical studies of OSS included approximately 20 % of patients ≥ 65 years old 16 19 , and we predicted that at least 10 % of patients in the general population will have renal or hepatic insufficiency 21 22 23 24 . This sample size gave a 2-sided 95 % level of confidence and a precision of ± 5 %. The target number of patients to be enrolled per site was initially set at 76 patients per site, based on the participation of 20 centres, but was later increased (up to 115 patients per site) to compensate for the inclusion of 16 centres.

The registry population was defined as all patients recruited, who at least partially took OSS and had available data about compliance without major deviation from the process of data collection. The safety population was all patients recruited, who at least partially took OSS and had follow-up safety information. The complete population consisted of all patients recruited, who at least partially took OSS, completed the colonoscopy and had available information on the cleansing of the colon without major deviation from the data collection process.

Descriptive statistics were used for continuous and categorical variables. Two-sided 95 % exact confidence intervals (CI; Clopper-Pearson) were calculated for binomial proportions, and two-sided 95 % confidence limits were calculated for the means. A paired student t-test was used to compare the mean percentage of compliance with the first and second dose (with a 5 % type I error rate). Analyses were conducted on the population as a whole and by dosing regimen, gender, country and special populations. Statistical analyses were performed by Biotrial Biometrics, France. Statistical evaluation was performed using Statistical Analysis System (SAS®; version 9.4 or higher).

## Results

### Patient disposition

**Fig. 1:**
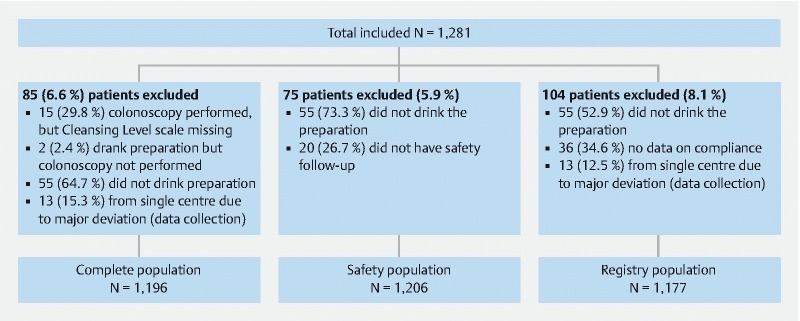
Patient flowchart.

Of 1,281 patients recruited from 16 centres ( **Appendix 1, Table A1** ), three in the Czech Republic (n = 236), eight in Germany (n = 678), two in the Netherlands (n = 171) and three in Poland (n = 196); 1,206 (94.1 %) patients constituted the safety population, 1,177 (91.9 %) the registry population, and 1,196 (93.4 %) the complete population ( Fig. 1 ). A total of 1,231 patients (96.1 %) completed the study (i. e. were treated and attended the second visit whether or not the colonoscopy was performed). In one centre, where there was evidence of major deviations in data collection, all patients (n = 13) were excluded from the registry population but included in the safety population.

### Baseline characteristics

Patient baseline characteristics and dosing regimens are shown in Table 1 . The overall mean age of patients was 59.2 years. The proportion of genders was well-balanced, but there were more male than female patients in the elderly subpopulation (54.2 % versus 45.8 %). The most frequent indications for OSS prior to colonoscopy were related to the presence or suspicion of CRC (57 %): screening (33.0 %) and a history of polyp or neoplasm (21.7 %). The proportion of patients in special populations (n = 544; 42.5 %) was higher than planned; however the majority were elderly (n = 502; 39.2 % of included population).

**Table 1:** Patient baseline characteristics and dosing regimen received (included population, n = 1281).

Baseline characteristics	n (%) 1				
Gender					
Male	647 (50.5)				
Female	634 (49.5)				
Age (years), mean (SD)	59.2 (13.5)				
Age category					
< 65 years	779 (60.8)				
≥ 65 years	502 (39.2)				
BMI (kg/m ^2^ ), mean (SD)	26.7 (4.8)				
Indication for colonoscopy					
Screening	423 (33.0)				
Polyp or neoplasm history	278 (21.7)				
Rectal bleeding	104 (8.1)				
Other GI bleeding	21 (1.6)				
Diarrhoea/constipation with unknown aetiology	115 (9.0)				
Abdominal pain	169 (13.2)				
Anaemia with unknown aetiology	31 (2.4)				
Inflammatory bowel disease	31 (2.4)				
Laser therapy	1 (0.1)				
Other 2	108 (8.4)				
Special populations					
Elderly patients ( ≥ 65 years)	502 (39.2)				
Suspicion of liver disease	31 (2.4)				
Hyperuricaemia or history of gout	52 (4.1)				
Renal insufficiency	11 (0.9)				
Inflammatory bowel disease	43 (3.4)				
Dosing regimen received 3	All	Czech Republic	Poland	Germany	Netherlands
Split dose	948 (74.2)	94 (39.8)	120 (62.2)	576 (85.0)	158 (92.4)
One day	252 (19.7)	133 (56.4)	63 (32.6)	56 (8.3)	0
Same day split dose	23 (1.8)	0	0	23 (3.4)	0
No treatment received	55 (4.3)	9 (3.8)	10 (5.2)	23 (3.4)	13 (7.6)

SD, standard deviation; BMI, body mass index; GI, gastrointestinal Split dose = the first dose is taken the day before procedure and the second dose on the day of procedure One day = both doses are taken the day before procedure Same day = both doses are taken on the day of the procedure

1 Results given in n (%), unless otherwise stated

2 Other; constipation/diarrhoea (11), weight loss (10), diverticular disease (9), dyspepsia (8), anaemia (8), polyp/cancer (8), follow-up colon cancer (6), abnormality imaging (6), hereditary syndrome (5), family history of colon cancer (4), screening (5), bleeding (2), unknown primary (2), abdominal pain (4), abdominal mass (1), colitis (3), primary sclerosing cholangitis (2), surveillance after liver transplantation (1), control post-surgery (benign) (3), anal pain (3), other bowel diseases (2), hepatopathy (2), nephrotic syndrome (1), dysphagia (1), irritable bowel syndrome (1), meteorism (1).

3 Data were missing from three patients. Percentages are stated as a proportion of the available 1278 patients

At baseline, laboratory values were available for 44.7 % of patients and 11.2 % of patients had an ECG. Most of the laboratory and ECG data came from seven and four centres, respectively. The laboratory parameter most frequently assessed at baseline was the international normalised ratio (INR; 27.9 %).

The proportion of patients who were prescribed a split dose or one day dose regimen varied between countries ( Table 1 ).

### Compliance with hydration guidelines

**Fig. 2:**
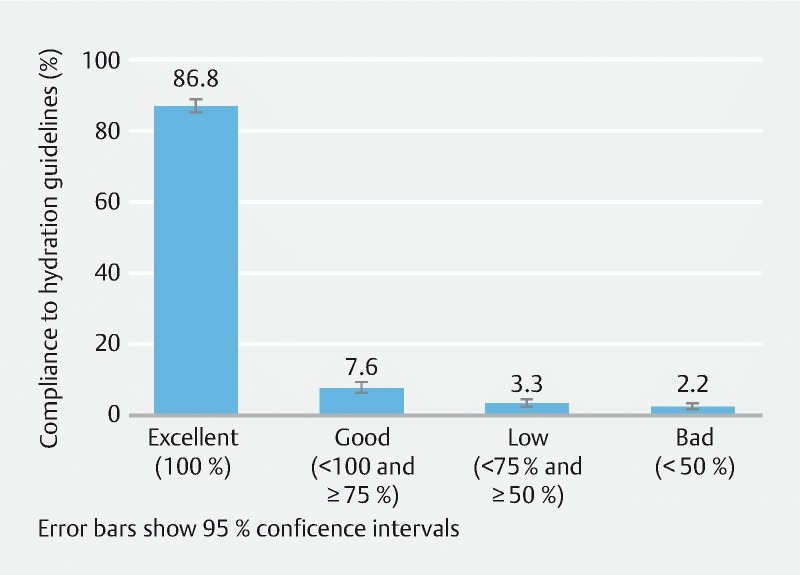
Patient compliance with hydration guidelines (registry population).

In the registry population most patients had a level of compliance with hydration assessed as excellent (1,022; 86.8 %) or good (90; 7.6 %), giving a total of 94.5 % (95 % CI: 93.0, 95.7) of patients with compliance ≥ 75 %, and 5.5 % (95 % CI: 4.3, 7.0) of patients with non-compliance ( Fig. 2 ). Data on hydration volume were missing for 43 patients (3.7 %) and were imputed as not having been taken; compliance with hydration was similar when these patients were excluded (96.6 % of patients with compliance ≥ 75 %, 95 % CI: 95.3, 97.5).

On average, patients drank 95.7 % (95 % CI: 94.9, 96.5) of the recommended 2,000 mL water or clear liquid. Significantly more liquid was taken with the first OSS dose than with the second dose ( *P* < 0.001), but the difference corresponded to 20 mL of clear liquid, which is not considered clinically significant. There was no difference in compliance with split dose (94.3 %; 95 % CI: 92.6, 95.7) or one-day dose (94.7 %; 95 % CI: 91.1, 97.1) regimens. There was no difference in compliance between males (95.0 %; 95 % CI: 93.0, 96.6) and females (93.9 % 95 % CI: 91.6, 95.7).

**Fig. 3:**
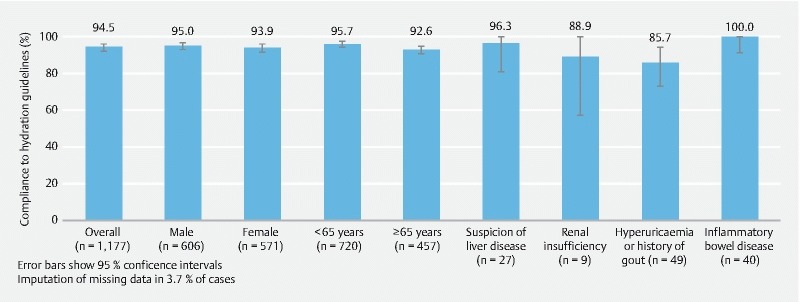
Patient compliance with hydration guidelines (compliance ≥ 75 %) among subgroups and special populations (registry population).

Compliance in the predefined special populations was similar to compliance in the registry population as a whole ( Fig. 3 ) but as the number of patients in the various special populations was small, conclusions can only be drawn for elderly patients. Most patients aged ≥ 65 years in the registry population had compliance assessed as excellent (386; 84.5 %) or good (37; 8.1 %), giving a total of 92.6 % (95 % CI: 89.8, 94.8) of elderly patients with compliance ≥ 75 %. No formal statistical comparison was made between compliance in patients < 65 years and ≥ 65 years. However, the 95 % CIs overlap and a significant difference is unlikely ( Fig. 3 ).

### Compliance with saline sulphate preparation

**Fig. 4:**
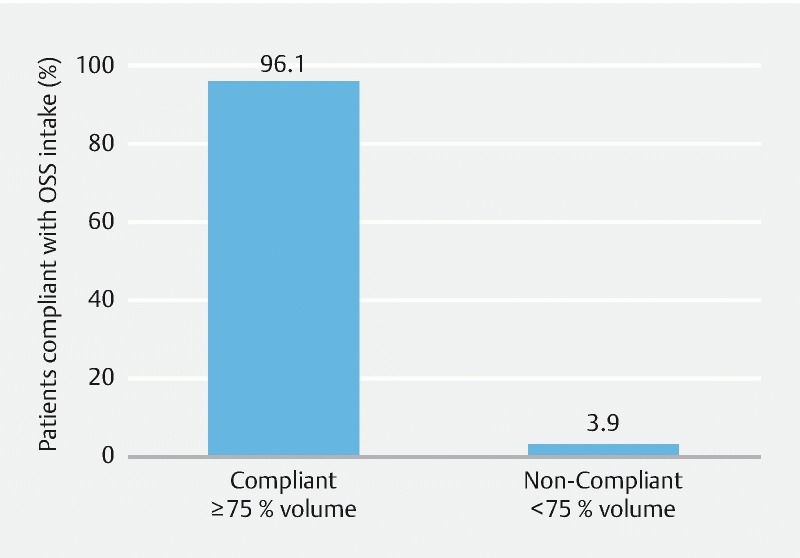
Patient compliance with OSS intake guidelines (registry population).

Overall, patients took an average of 96.8 % of the recommended volume of the preparation. However, 46 patients (3.9 %) were considered non-compliant having taken < 75 % of the recommended volume ( Fig. 4 ). A total of 1,131 patients (96.1 %) were compliant with the saline sulphate preparation intake, taking ≥ 75 % of the recommended volume ( Fig. 4 ).

### Cleansing level

**Fig. 5:**
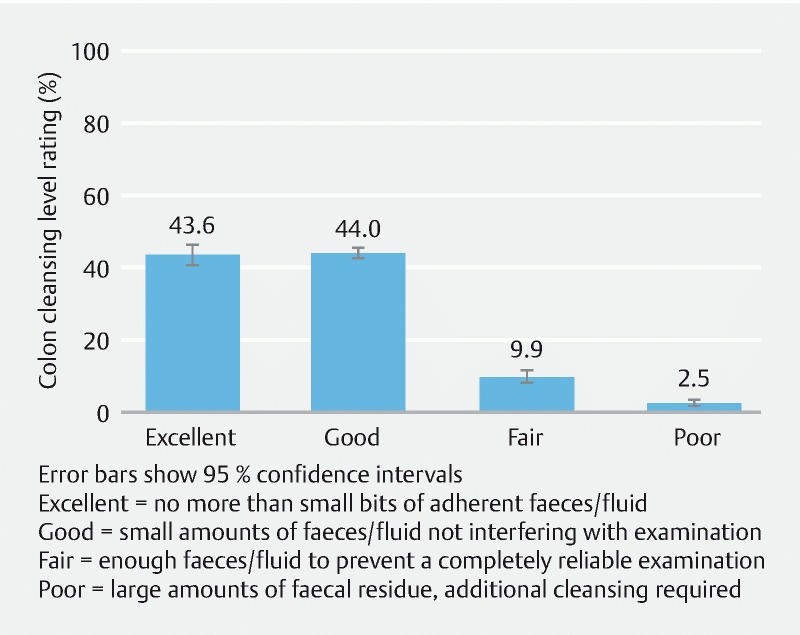
Investigator-reported colon cleansing level (complete population).

The cleansing level of the colon at the colonoscopy was considered excellent or good in 87.6 % of the complete population (good 44.0 %; 95 % CI: 41.1, 46.8. Excellent 43.6 %; 95 % CI: 40.8, 46.5; Fig. 5 ).

### Safety

A total of 374 participants in the safety population (31.0 %) reported 885 treatment-emergent AEs (TEAEs); 27.3 % of the safety population reported 758 TEAEs considered related to the treatment ( Table 2 ). The frequency of TEAEs was greater in females than males ( Table 2 ). This difference was not associated with country, age, indication for colonoscopy or rapidity of intake. Nausea (irrespective of relatedness to treatment) was reported by 21.6 % (95 % CI: 18.5, 25.1) of females and 7.4 % (95 % CI: 5.5, 9.8) of males. The nature and intensity of TEAEs were similar in both genders ( Table 2 ).

**Table 2:** Patients reporting treatment-emergent adverse events (safety population).

	All (n = 1206)	Males (n = 618)	Females (n = 588)
	n (%) [95 % CI]		
**Summary of safety**			
Any TEAEs	374 (31.3) [28.4, 33.7]	128 (20.7) [17.6, 24.1]	246 (41.8) [37.8, 45.9]
Any serious TEAEs	2 (0.2) [0.0, 0.6]	1 (0.2) [0.0, 0.9]	1 (0.2) [0.0, 0.9]
Any related TEAEs ^*^	329 (27.3) [24.8, 29.9]	110 (17.8) [14.9, 21.0]	219 (37.2) [33.3, 41.3]
Intensity of related TEAEs			
Severe intensity	23 (1.9) [1.2, 2.8]	8 (1.3) [0.6, 2.5]	15 (2.6) [1.4, 4.2]
Moderate intensity	91 (7.5) [6.1, 9.2]	29 (4.7) [3.2, 6.7]	62 (10.5) [8.2, 13.3]
Mild intensity	263 (21.8) [1.0, 2.4]	92 (14.9) [12.2, 17.9]	171 (29.1) [25.4, 32.9]
Missing intensity	18 (1.5) [0.9, 2.3]	3 (0.5) [0.1, 1.4]	15 (2.6) [1.4, 4.2]
**Most frequent TEAEs (≥ 1 % patients safety population)**			
**Gastrointestinal disorders**	296 (24.5) [22.1, 27.1]	94 (15.2) [12.5, 18.3]	202 (34.4) [30.5, 38.3]
Nausea	173 (14.3) [12.4, 16.5]	46 (7.4) [5.5, 9.8]	127 (21.6) [18.3, 25.1]
Abdominal pain	54 (4.5) [3.5, 5.8]	22 (3.6) [2.2, 5.3]	32 (5.4) [3.8, 7.6]
Abdominal pain upper	46 (3.8) [2.8, 5.1]	12 (1.9) [1.0, 3.4]	34 (5.8) [4.0, 8.0]
Abdominal distension	38 (3.2) [2.2, 4.3]	9 (1.5) [0.7, 2.7]	29 (4.9) [3.3, 7.0]
Vomiting	38 (3.2) [2.2, 4.3]	7 (1.1) [0.5, 2.3]	31 (5.3) [3.6, 7.4]
Eructation	20 (1.7) [1.0, 2.5]	4 (0.6) [0.2, 1.6]	16 (2.7) [1.6, 4.4]
Flatulence	15 (1.2) [0.7, 2.0]	5 (0.8) [0.3, 1.9]	10 (1.7) [0.8, 3.1]
Anorectal discomfort	14 (1.2) [0.6, 1.9]	2 (0.3) [0.0, 1.2]	12 (2.0) [1.1, 3.5]
Abdominal discomfort	12 (1.0) [0.5, 1.7]	6 (1.0) [0.4, 2.1]	6.0 (1.0) [0.4, 2.2]
**Nervous system disorders**	112 (9.3) [7.7, 11.1]	36 (5.8) [4.1, 8.0]	76 (12.9) [10.3, 15.9]
Headache	91 (7.5)[106] [6.1, 9.2]	30 (4.9) [3.3, 6.9]	61 (10.4) [8.0, 13.1]
Dizziness	21 (1.7) [1.1, 2.6]	6 (1.0) [0.4, 2.1]	15 (2.6) [1.4, 4.2]
**General disorders and administration site conditions**	69 (5.7) [4.5, 7.2]	20 (3.2) [2.0, 5.0]	49 (8.3) [6.2, 10.9]
Chills	36 (3.0) [2.1, 4.1]	10 (1.6) [0.8, 3.0]	26 (4.4) [2.9, 6.4]
Feeling cold	16 (1.3) [0.8, 2.1]	6 (1.0) [0.0, 0.9]	10 (1.7) [0.8, 3.1]
Malaise	13 (1.1) [0.6, 1.8]	1 (0.2) [0.0, 0.9]	12 (2.0) [1.1, 3.5]
**Ear and labyrinth disorders**	16 (1.3) [0.8, 2.1]	6 (1.0) [0.4, 2.1]	10 (1.7) [0.8, 3.1]
Vertigo	16 (1.3) [0.8, 2.1]	6 (1.0) [0.4, 2.1]	10 (1.7) [0.8, 3.1]

CI, confidence interval; TEAE, treatment-emergent adverse event

Likewise, the most frequent treatment-related TEAEs were gastrointestinal disorders (reported in 273/1,206 patients in the safety population; 22.6 %), including nausea (162/1206: 13.4 %) and abdominal pain (48/1,206: 4.0 %) ( Table 3 ). The only other treatment-related TEAE occurring in more than 5 % of patients was headache (71/1,206: 5.9 %). Vomiting occurred in 37 patients (3.1 %). The percentage of patients reporting treatment-related TEAEs was similar among non-compliant and compliant patients; 25/67 (29.1 %, 95 % CI: 19.8, 39.9) and 304/1,120 (27.1 %, 95 % CI: 24.6, 29.8), respectively. AEs possibly indicative of dehydration, including headaches, dry mouth, thirst, dizziness, cardiovascular disorder, palpitation, tachycardia, hypotension, malaise and vertigo, were reported in 137/1,206 (11.4 %) patients. Compliance with hydration guidelines in these 137 patients was similar to the registry population (92.7 % of patients with compliance ≥ 75 %).

**Table 3:** Number (%) of patients reporting treatment-emergent adverse events by relationship to treatment where total number of events was > 1 % (safety population).

Primary system organ class	Related	Not related
	n (%)	
**Any TEAEs**	329 (27.3)	74 (6.1)
**Gastrointestinal disorders**	273 (22.6)	36 (3.0)
Nausea	162 (13.4)	11 (0.9)
Abdominal pain	48 (4.0)	6 (0.5)
Abdominal pain upper	44 (3.6)	2 (0.2)
Abdominal distension	35 (2.9)	3 (0.2)
Vomiting	37 (3.1)	1 (0.1)
Eructation	17 (1.4)	3 (0.2)
Flatulence	12 (1.0)	3 (0.2)
**Nervous system disorders**	87 (7.2)	26 (2.2)
Headache	71 (5.9)	20 (1.7)
Dizziness	13 (1.1)	8 (0.7)
**General disorders and administration site conditions**	51 (4.2)	18 (1.5)
Chills	27 (2.2)	9 (0.7)
Feeling cold	12 (1.0)	4 (0.3)
Malaise	13 (1.1)	0
**Ear and labyrinth disorders**	16 (1.3)	0
Vertigo	16 (1.3)	0

CI, confidence interval; TEAE, treatment-emergent adverse event

Acute/immediate TEAEs occurring in the predefined special populations were mainly gastrointestinal in nature and were mild. The nature, frequency and intensity of treatment-related TEAEs were similar in patients < 65 (244/738 [33.1 %]; 95 % CI 29.7, 36.6) and ≥ 65 years of age (134/468 [28.6 %]; 95 % CI [24.6, 33.0]). There was no significant difference between the AE profile observed in special populations compared with the overall study population.

One death occurred due stage IV ovarian cancer and urosepsis in a patient with chronic kidney disease, which was not considered related to study medication or procedure. Two patients experienced SAEs; a hypersensitivity reaction to a nitroglycerin spray, and a post-procedural haemorrhage. These two events were not related to OSS and both patients recovered from the events.

## Discussion

The primary objective of this non-interventional study was to document non-compliance with the OSS hydration guidelines in real-life clinical practice. A total of 94.5 % had good-to-excellent compliance and 5.5 % were non-compliant with the recommended liquid intake. As patients with missing data were classified as non-compliant, actual compliance to hydration guidelines may be higher than 94.5 %. On average patients took 96.8 % of the preparation. This is similar to previously reported compliance rates in interventional trials of OSS 16 19 . The high level of compliance with the hydration guidelines and whole preparation confirms that OSS is used according to the prescribing information in real-life settings. Considering that preparation palatability is an important factor limiting compliance, ingestion of the saline preparation was accepted by most patients, with only 46 patients (3.9 %, 95 % CI: 2.9; 5.2) ingesting less than 75 % of the preparation.

There were no differences in compliance with hydration according to gender or regimen (split dose or one day dose), and the slightly lower compliance in elderly patients than those < 65 years of age (both groups had compliance > 90 %) was not statistically or clinically significant.

This clinical importance of good compliance was reflected in the excellent or good colon cleansing scores (87.6 %). In the non-interventional setting of this study, and considering that 19.7 % of patients took OSS as a one-day administration, this level of colon cleansing should be considered satisfactory. It should be noted that a validated cleansing efficacy assessment was not a primary goal of this study.

In this real-life setting, the safety profile of OSS was similar to that in the prescribing information. OSS was administered as two separate doses both in split and in non-split regimens. When patients experienced the same AE twice (once per dose), then these were counted as two separate events. Consequently, the percentage of AEs reported in this study is higher than other previously reported studies 16 . However, treatment-related TEAEs in this study did not differ in nature or in intensity from the known safety profile of OSS. Most TEAEs were gastrointestinal, which may partially explain the slightly lower compliance with the second dose of water or saline solution.

Female patients reported twice as many TEAEs as males, but their nature and intensity were similar in both groups. This difference was not explained by other factors, including age, country, indication for colonoscopy or rapidity of intake of the preparation. Worse tolerability of colonoscopy preparations in females than in males has been reported in previous studies involving OSS 25 and other bowel preparations 26 .

While patients identified as non-compliant with hydration or at risk for electrolyte shifts did not experience more TEAEs, or TEAEs of a different nature, compared to the rest of the population, their proportion was small compared to the overall population of the study. OSS is contraindicated in patients with advanced renal, liver or heart failure. These patients could receive OSS (in line with the Summary of Product Characteristics 15 ) after baseline and post-treatment tests to evaluate the electrolyte status and renal function. However, in this non-interventional study using a newly considered compound, it is likely that investigators did not include more fragile patients even if eligible for OSS. In addition, laboratory tests were not required and patients with more moderate renal disease, for example, may not have been identified, and complete medical histories were not available for all patients to the investigator. Thus, the true population of patients at risk for electrolyte shifts may have been greater than those captured in the reported special populations. Nevertheless, no firm conclusion regarding the presence or absence of electrolyte shifts or acute AEs in these populations can be drawn.

AEs possibly indicative of clinical dehydration did not occur more frequently in compliant or non-compliant patients. However, this study was non-interventional and laboratory data was not available. Electrolyte abnormalities are a risk with all bowel preparations, but in a recent US health database study conducted on screening colonoscopies, their incidence was reported to be significantly lower with the use of OSS than with other bowel preparations 27 .

The study results can be generalized to the European population exposed to the product, but several limitations should be underlined. Country selection was based on product availability at the time of study initiation, and half of the study population was recruited at German sites. There were disparities in access to care, or absence of reimbursement of the drug in some of the study countries. These reflect economic disparities between the different European countries where the product is available. As this non-interventional study was designed to include randomly selected centres, many of which did not typically conduct clinical trials, missing information on medical history and laboratory values has an impact on some conclusions. However, these limitations do not impact generalisability of the study conclusions.

## Conclusions

In this non-interventional study, treatment compliance with hydration guidelines for bowel cleansing preparation OSS was excellent or good in 94.5 % of patients. Subsequent colon cleaning scores were good-to-excellent in 87.6 % of patients.

Overall and in special populations, OSS was well tolerated and the safety profile was similar to previous reports.

## Supplementary material

Supplementary material Zusatzmaterial

## References

[1] Hassan C Bretthauer M Kaminski M F Bowel preparation for colonoscopy: European Society of Gastrointestinal Endoscopy (ESGE) guideline Endoscopy 2013 45 142 150 23335011 10.1055/s-0032-1326186

[2] Menees S B Kim H M Wren P Patient compliance and suboptimal bowel preparation with split-dose bowel regimen in average-risk screening colonoscopy Gastrointest Endosc 2014 79 811 820 e813 24631492 10.1016/j.gie.2014.01.024 PMC4107415

[3] Beck D E Bowel preparation for colonoscopy Clin Colon Rectal Surg 2010 23 10 13 21286285 10.1055/s-0030-1247851 PMC2850161

[4] American Society of Colon Rectal Surgeons American Society for Gastrointestinal Endoscopy Society of American Gastrointestinal Endoscopic Surgeons A consensus document on bowel preparation before colonoscopy: prepared by a Task Force from the American Society of Colon and Rectal Surgeons (ASCRS), the American Society for Gastrointestinal Endoscopy (ASGE), and the Society of American Gastrointestinal and Endoscopic Surgeons (SAGES) Surg Endosc 2006 20 1161 16799744 10.1007/s00464-006-3037-1

[5] Harewood G C Sharma V K de Garmo P Impact of colonoscopy preparation quality on detection of suspected colonic neoplasia Gastrointest Endosc 2003 58 76 79 12838225 10.1067/mge.2003.294

[6] Lebwohl B Kastrinos F Glick M The impact of suboptimal bowel preparation on adenoma miss rates and the factors associated with early repeat colonoscopy Gastrointest Endosc 2011 73 1207 1214 21481857 10.1016/j.gie.2011.01.051 PMC3106145

[7] Ness R M Manam R Hoen H Predictors of inadequate bowel preparation for colonoscopy Am J Gastroenterol 2001 96 1797 1802 11419832 10.1111/j.1572-0241.2001.03874.x

[8] Chung Y W Han D S Park K H Patient factors predictive of inadequate bowel preparation using polyethylene glycol: a prospective study in Korea J Clin Gastroenterol 2009 43 448 452 18978506 10.1097/MCG.0b013e3181662442

[9] Govani S M Elliott E E Menees S B Predictors of suboptimal bowel preparation in asymptomatic patients undergoing average-risk screening colonoscopy World J Gastrointest Endosc 2016 8 616 622 27668072 10.4253/wjge.v8.i17.616 PMC5027032

[10] Harewood G C Wiersema M J Melton L J A prospective, controlled assessment of factors influencing acceptance of screening colonoscopy Am J Gastroenterol 2002 97 3186 3194 12492209 10.1111/j.1572-0241.2002.07129.x

[11] Kilgore T W Abdinoor A A Szary N M Bowel preparation with split-dose polyethylene glycol before colonoscopy: a meta-analysis of randomized controlled trials Gastrointest Endosc 2011 73 1240 1245 21628016 10.1016/j.gie.2011.02.007

[12] Seo E H Kim T O Park M J Optimal preparation-to-colonoscopy interval in split-dose PEG bowel preparation determines satisfactory bowel preparation quality: an observational prospective study Gastrointest Endosc 2012 75 583 590 22177570 10.1016/j.gie.2011.09.029

[13] Marmo R Rotondano G Riccio G Effective bowel cleansing before colonoscopy: a randomized study of split-dosage versus non-split dosage regimens of high-volume versus low-volume polyethylene glycol solutions Gastrointest Endosc 2010 72 313 320 20561621 10.1016/j.gie.2010.02.048

[14] World Health Organization Declaration of Helsinki: Ethical Principles for Medical Research Involving Human Subjects Bulletin of the World Health Organization 2001 79 373 374 11357217 PMC2566407

[15] Izinova concentrate for oral solution. Summary of Product Characteristics 2015

[16] Di Palma J A Rodriguez R McGowan J A randomized clinical study evaluating the safety and efficacy of a new, reduced-volume, oral sulfate colon-cleansing preparation for colonoscopy Am J Gastroenterol 2009 104 2275 2284 19584830 10.1038/ajg.2009.389

[17] Di Palma J A Wolff B G Meagher A Comparison of reduced volume versus four liters sulfate-free electrolyte lavage solutions for colonoscopy colon cleansing Am J Gastroenterol 2003 98 2187 2191 14572566 10.1111/j.1572-0241.2003.07690.x

[18] Di Palma J A McGowan J Cleveland M V Clinical trial: an efficacy evaluation of reduced bisacodyl given as part of a polyethylene glycol electrolyte solution preparation prior to colonoscopy Aliment Pharmacol Ther 2007 26 1113 1119 17894653 10.1111/j.1365-2036.2007.03459.x

[19] Rex D K DiPalma J A McGowan J A comparison of oral sulfate solution with sodium picosulfate: magnesium citrate in split doses as bowel preparation for colonoscopy Gastrointest Endosc 2014 80 1113 1123 25028274 10.1016/j.gie.2014.05.329

[20] Rex D K Di Palma J A Rodriguez R A randomized clinical study comparing reduced-volume oral sulfate solution with standard 4-liter sulfate-free electrolyte lavage solution as preparation for colonoscopy Gastrointest Endosc 2010 72 328 336 20646695 10.1016/j.gie.2010.03.1054

[21] Hill N R Fatoba S T Oke J L Global Prevalence of Chronic Kidney Disease - A Systematic Review and Meta-Analysis PLoS One 2016 11 e0158765 27383068 10.1371/journal.pone.0158765 PMC4934905

[22] Pimpin L Cortez-Pinto H Negro F Burden of liver disease in Europe: Epidemiology and analysis of risk factors to identify prevention policies J Hepatol 2018 69 718 735 29777749 10.1016/j.jhep.2018.05.011

[23] Blachier M Leleu H Peck-Radosavljevic M The burden of liver disease in Europe: a review of available epidemiological data J Hepatol 2013 58 593 608 23419824 10.1016/j.jhep.2012.12.005

[24] Sarin S Maiwall R Global burden of liver disease: a true burden on health sciences and economiesWorld Gastroenterology Organanisation2012 17

[25] Holt E W Yimam K K Ma H Patient tolerability of bowel preparation is associated with polyp detection rate during colonoscopy J Gastrointestin Liver Dis 2014 23 135 140 24949604 10.15403/jgld.2014.1121.232.ewh1

[26] Lawrance I C Willert R P Murray K A validated bowel-preparation tolerability questionnaire and assessment of three commonly used bowel-cleansing agents Dig Dis Sci 2013 58 926 935 23095990 10.1007/s10620-012-2449-0

[27] Anastassopoulos K Farraye F A Knight T A comparative study of treatment-emergent adverse events following use of common bowel preparations among a colonoscopy screening population: results from a post-marketing observational study Dig Dis Sci 2016 61 2993 3006 27278957 10.1007/s10620-016-4214-2 PMC5020112

