# Supplementary material for: A European, multicentre, observational, post-authorisation safety study of oral sulphate solution: compliance and safety
Source: Endosc Int Open. 2020 Feb 21;8(3):E247–56. doi: 10.1055/a-1090-7289 (PMC7055619; doi:10.1055/a-1090-7289)
Supplement: Supplementary file 1 — Supplementary material [file 1659supmat-10-1055-a-1090-7289.pdf]

Appendix 1 - Tables

Table A1. Participating centres

**Czech Republic**
Ustredni vojenska nemocnice<br>Oddeleni gastrointestinalni endoskopie<br>U Vojenske nemocnice 1200<br>169 02 Praha 6
Krajská zdravotní, a.s., nemocnice Teplice, Gastroenterologie<br>Duchcovská 53<br>415 01 Teplice
Ústeckoorlická nemocnice, a.s. Endoskopie<br>ČSA 1076<br>562 01 Ústí nad Orlicí
**Germany**
Klinikum Aschaffenburg-Alzenau, Standort Aschaffenburg<br>Am Hasenkopf<br>63739 Aschaffenburg
Gastro-Praxis Wiesbaden (Gastrodata)<br>Langenbeckplatz 2<br>65189 Wiesbaden
Praxisgemeinschaft Innere am Stadtpark<br>Am Stadtpark 2<br>90409 Nürnberg
MVZ Innere Medizin Marburg, Dres. Drude und Partner<br>Biegenstr. 3<br>35037 Marburg
Medizinisches Versorgungszentrum Portal 10<br>Albersloher Weg 10<br>48155 Münster
Chefarzt der Medizinischen Klinik III, Westpfalz Klinikum GmbH, Standort I Kaiserslautern<br>Hellmut-Hartert-Str. 1,<br>67655 Kaiserslautern
MVZ Ortenau Achern, Innere Medizin<br>Josef-Wurzler-Str. 7/3,<br>77855 Achern
Gemeinschaftspraxis Dres. Klausmann<br>Elisenstr. 28<br>63739 Aschaffenburg
**The Netherlands**
Erasmus MC<br>Maag-Darm en Leverziekte

's Gravendijkwal 230<br>3015 CE Rotterdam<br><br>Elisabeth-Tweesteden Ziekenhuis<br>Dr. Deelenlaan 5<br>5042 AD Tilburg
**Poland**<br><br>Maria Sklodowska-Curie Institute — Oncology Center<br>Roentgen 5<br>02-781 Warsaw<br><br>Copernicus Medical Entity<br>Al. Jana Pawła II 50<br>80-462 Gdańsk<br><br>Instytut Medycyny<br>Jaczewskiego 2<br>20-090 Lublin

Appendix 2 – Patient leaflet (next page and following)

This instruction form is provided by the company which markets IZINOVA to the health care professionals so they can inform their patients on the day of the prescription, in accordance with the risk management plan in force for this medicine

To be filled in by the health care professional

IZINOVA

concentrate for oral solution
Sodium **sulphate** anhydrous,
magnesium **sulphate** heptahydrate
and potassium **sulphate**

PATIENT REGIMEN (tick recommended plan):

☐ **TWO DAY (Split Dose) PLAN**

1st dose the day BEFORE procedure; 2nd dose the day of procedure

☐ **ONE DAY PLAN**

2 doses the day BEFORE procedure

Your procedure will be performed by Doctor: ______________________________

Date of procedure: ______________________________ Arrive at: ______________ AM / PM

Location: ______________________________

How to Get Prepared for the Procedure

To ensure the effectiveness of the bowel cleansing and therefore the success of the procedure, it is essential that you strictly comply with the following instructions:

- On the day before the procedure, you may have a light breakfast; afterwards you should **ONLY** have clear liquids for lunch, dinner and any other meals until the procedure is performed.
- The preparation with IZINOVA, concentrate for oral solution (sodium sulphate anhydrous, magnesium sulphate heptahydrate and potassium sulphate) consists of 2 dose intakes according to the following sequence: →

Importance of Appropriate Hydration

Since diarrhoea induces dehydration, it is very important that you strictly follow the above instructions and drink as much additional clear liquid as necessary to maintain an appropriate level of hydration.

✔ **You can drink any of the following clear liquids:**

Clear fluids such as water, tea or coffee (no milk or non dairy creamers). Sweeteners are acceptable; carbonated or non carbonated soft drinks or strained fruit juices **without** pulp (non coloured red or purple), clear soups or soups strained to remove any solids.

✘ **DO NOT drink:**

Milk, anything coloured red or purple, alcoholic beverages, fruit juices or soft drinks **with** pulp

FIRST DOSE:

begin the preparation on ________________(Date)
at ______________PM and proceed as shown below:

1

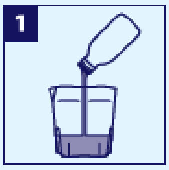

Pour the contents of one bottle of IZINOVA into the cup.

2

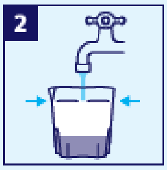

Add water to the medicine until the level reaches the line on the cup.

3

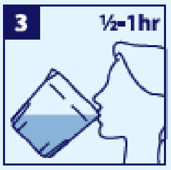

Take your time (over half an hour to an hour) to drink all the liquid in the cup.

4

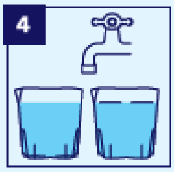

IMPORTANT
Drink two (2) more cups of water (or authorised clear liquids - *see authorised drinks)* over the next hour

SECOND DOSE:

begin the preparation on ________________ (Date)
at ____________ **AM / PM** and proceed as shown above

For the second dose, repeat steps **1 through 4** shown above using the other bottle.

Note: You **must** finish drinking the final cup of water or clear liquid at least 1 hour before your procedure.

TURN OVER →

This instruction form is provided by the company which markets IZINOVA to the health care professionals so they can inform their patients on the day of the prescription, in accordance with the risk management plan in force for this medicine

IZINOVA

concentrate for oral solution
Sodium **sulphate** anhydrous,
magnesium **sulphate** heptahydrate
and potassium **sulphate**

To be filled in by the patient

MY RECOMMENDED REGIMEN:

☐ **TWO DAY (Split Dose) PLAN**
1st dose the day BEFORE procedure; 2nd dose the day of procedure

☐ **ONE DAY PLAN**
2 doses the day BEFORE procedure

PLEASE READ THE PATIENT LEAFLET CAREFULLY BEFORE TAKING THIS MEDICINE

Do not forget to fill in this form and bring it back to your Doctor on the day of the Procedure

The table below will help you check that you have appropriately followed the instructions and will ensure a good quality preparation for your Doctor.
Please note the date of administration and the hours of the different intakes identified in the table.

You should do your best to drink all the preparation and the additional liquid.

FIRST DOSE: Date: ___________________________

You should do your best to drink all the preparation

STEP		Time of Intake
Start of treatment - STEPS 1 - 3<br><br>Contents of the first bottle of IZINOVA diluted **in water** into the cup until the level reaches the line of the cup	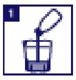<br>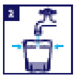<br>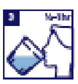	Time of intake of the **first** dose of diluted Izinova:<br><br>______________*PM*
Additional intake of water or clear liquids - STEP 4<br><br>2 x the cup filled with water or authorised clear liquids	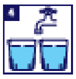	Time of intake of the **first** cup of water (or clear liquids):<br>______________*PM*<br><br>Time of intake of the **second** cup of water (or clear liquids):<br>______________*PM*

DID YOU DRINK ALL OF THE PREPARATION?

☐ Yes ☐ No

If NO approximate quantity taken:

____

**clear liquids consumed to ensure appropriate hydration**	
Nature of liquid(s)	Amount
	Did you drink all of the cup content?<br>☐ YES ☐ NO
	☐ YES ☐ NO

SECOND DOSE: Date: ___________________________

You should do your best to drink all the preparation

STEP		Time of Intake
Second sequence of the treatment - REPEAT STEPS 1 - 3<br><br>Contents of the second bottle of Izinova diluted **in water** into the cup until the level reaches the line of the cup	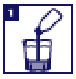<br>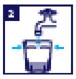<br>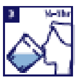	Time of intake of the second dose of diluted Izinova<br><br>______________*AM / PM*
Additional intake of water or clear liquids - STEP 4<br><br>2 x the cup filled with water or authorised clear liquids	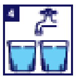	Time of intake of the **first** cup of water (or clear liquids):<br>______________*AM / PM*<br><br>Time of intake of the **second** cup of water (or clear liquids):<br>______________*AM / PM*

DID YOU DRINK ALL OF THE PREPARATION?

☐ Yes ☐ No

If NO approximate quantity taken:

____

**clear liquids consumed to ensure appropriate hydration**	
Nature of liquid(s)	Amount
	Did you drink all of the cup content?<br>☐ YES ☐ NO
	☐ YES ☐ NO

Subject number |___|___|___|___|___|___|/ |___|___|___|___|___|

FIRST DOSE OF IZINOVA®/EZICLEN®

<table>
<tr><th>Date of intake</th><th>Procedure</th><th>Time of intake
(24 hours clock)
E.g.: 22 h 35 min</th><th>Specify the volume of solution/liquids you've managed to drink</th><th>Specify the nature of liquids taken
(Water, tea, coffee, clear soup...):</th></tr>
<tr><td>|__|__|/|__|__|/201|__|
d d m m y y y y</td><td>First
IZINOVA®/EZICLEN®dose
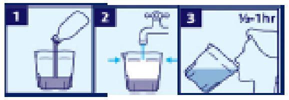</td><td>Start time
|__|__| h |__|__| min
Stop time
|__|__| h |__|__| min</td><td>❍ The entire cup
❍ Between half of the cup and the entire cup
❍ Less than half cup
❍ No intake</td><td></td></tr>
<tr><td rowspan="2">Additional intake of liquids
(water or clear liquids)</td><td>1st cup
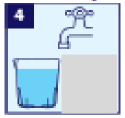</td><td>Start time (1st cup)
|__|__| h |__|__| min</td><td>❍ The entire cup
❍ Between half of the cup and the entire cup
❍ Less than half cup
❍ No intake</td><td></td></tr>
<tr><td>2nd cup
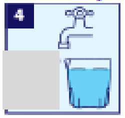</td><td>Start time (2nd cup)
|__|__| h |__|__| min</td><td>❍ The entire cup
❍ Between half of the cup and the entire cup
❍ Less than half cup
❍ No intake</td><td></td></tr>
</table>

Turn the page and specify the type and quantity of food eaten during this first step of the preparation, as well as any experienced side effects. ⇨

Thank you for completing this form. Don't forget to bring it back on the day of the procedure.

Subject number |___|___|___|___|___|___|/ |___|___|___|___|___|

FIRST DOSE OF IZINOVA®/EZICLEN®

DIET: specify the type and the amount of food eaten.

Time of intake	Type and quantity *(i.e. one hard-boiled egg)*
|__|__| h |__|__| min	....
|__|__| h |__|__| min	....
|__|__| h |__|__| min	....

After the first dose, have you experienced any side effects? ☐ Yes ☐ No

↳ If yes, please provide as much information as you can below and share this information with your Doctor accordingly on the day of the procedure

Thank you for completing this form. Don’t forget to bring it back on the day of the procedure.

Subject number |___|___|___|___|___|___|/ |___|___|___|___|___|

SECOND DOSE OF IZINOVA®/EZICLEN®

<table>
<tr><th>Date of intake</th><th>Procedure</th><th>Time of intake
(24 hours clock)
E.g.: 22 h 35 min</th><th>Specify the volume of solution/liquids you've managed to drink</th><th>Specify the nature of liquids taken
(Water, tea, coffee, clear soup…):</th></tr>
<tr><td>|__|__|/|__|__|/201|__|
d d m m y y y y</td><td>Second IZINOVA®/EZICLEN®dose
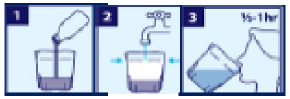</td><td>Start time
|__|__| h |__|__| min
Stop time
|__|__| h |__|__| min</td><td>❍ The entire cup
❍ Between half of the cup and the entire cup
❍ Less than half cup
❍ No intake</td><td></td></tr>
<tr><td rowspan="2">Additional intake of liquids
(water or clear liquids)</td><td>1st cup
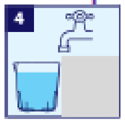</td><td>Start time (1st cup)
|__|__| h |__|__| min</td><td>❍ The entire cup
❍ Between half of the cup and the entire cup
❍ Less than half cup
❍ No intake</td><td></td></tr>
<tr><td>2nd cup
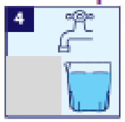</td><td>Start time (2nd cup)
|__|__| h |__|__| min</td><td>❍ The entire cup
❍ Between half of the cup and the entire cup
❍ Less than half cup
❍ No intake</td><td></td></tr>
</table>

Turn the page and specify the type and quantity of food eaten during this second step of the preparation, as well as any experienced side effects. ⇨

Thank you for completing this form. Don't forget to bring it back on the day of the procedure.

Subject number |___|___|___|___|___|___|/ |___|___|___|___|___|

SECOND DOSE OF IZINOVA®/EZICLEN®

DIET: specify the type and the amount of food eaten.

Time of intake	Type and quantity *(i.e. one hard-boiled egg)*
|__|__| h |__|__| min	....
|__|__| h |__|__| min	....
|__|__| h |__|__| min	....

After the second dose, have you experienced any side effects? ☐ Yes ☐ No

↳ If yes, please provide as much information as you can below and share this information with your Doctor accordingly on the day of the procedure

Thank you for completing this form. Don't forget to bring it back on the day of the procedure.
